# Formative Perceptions of a Digital Pill System to Measure Adherence to Heart Failure Pharmacotherapy: Mixed Methods Study

**DOI:** 10.2196/48971

**Published:** 2024-02-15

**Authors:** Peter R Chai, Jenson J Kaithamattam, Michelle Chung, Jeremiah J Tom, Georgia R Goodman, Mohammad Adrian Hasdianda, Tony Christopher Carnes, Muthiah Vaduganathan, Benjamin M Scirica, Jeffrey L Schnipper

**Affiliations:** 1 Department of Emergency Medicine Brigham and Women’s Hospital Boston, MA United States; 2 Department of Psychosocial Oncology and Palliative Care Dana Farber Cancer Institute Boston, MA United States; 3 The Koch Institute for Integrated Cancer Research Massachusetts Institute of Technology Cambridge, MA United States; 4 The Fenway Institute Boston, MA United States; 5 eTectRx Gainesville, FL United States; 6 Division of Cardiovascular Medicine Department of Medicine Brigham and Women's Hospital Boston, MA United States; 7 Division of Hospital Medicine, Department of Medicine Brigham and Women’s Hospital Boston, MA United States

**Keywords:** behavioral interventions, cardiac treatment, digital pill system, heart failure medication, heart failure, ingestible sensors, medication adherence

## Abstract

**Background:**

Heart failure (HF) affects 6.2 million Americans and is a leading cause of hospitalization. The mainstay of the management of HF is adherence to pharmacotherapy. Despite the effectiveness of HF pharmacotherapy, effectiveness is closely linked to adherence. Measuring adherence to HF pharmacotherapy is difficult; most clinical measures use indirect strategies such as calculating pharmacy refill data or using self-report. While helpful in guiding treatment adjustments, indirect measures of adherence may miss the detection of suboptimal adherence and co-occurring structural barriers associated with nonadherence. Digital pill systems (DPSs), which use an ingestible radiofrequency emitter to directly measure medication ingestions in real-time, represent a strategy for measuring and responding to nonadherence in the context of HF pharmacotherapy. Previous work has demonstrated the feasibility of using DPSs to measure adherence in other chronic diseases, but this strategy has yet to be leveraged for individuals with HF.

**Objective:**

We aim to explore through qualitative interviews the facilitators and barriers to using DPS technology to monitor pharmacotherapy adherence among patients with HF.

**Methods:**

We conducted individual, semistructured qualitative interviews and quantitative assessments between April and August 2022. A total of 20 patients with HF who were admitted to the general medical or cardiology service at an urban quaternary care hospital participated in this study. Participants completed a qualitative interview exploring the overall acceptability of and willingness to use DPS technology for adherence monitoring and perceived barriers to DPS use. Quantitative assessments evaluated HF history, existing medication adherence strategies, and attitudes toward technology. We analyzed qualitative data using applied thematic analysis and NVivo software (QSR International).

**Results:**

Most participants (12/20, 60%) in qualitative interviews reported a willingness to use the DPS to measure HF medication adherence. Overall, the DPS was viewed as useful for increasing accountability and reinforcing adherence behaviors. Perceived barriers included technological issues, a lack of need, additional costs, and privacy concerns. Most were open to sharing adherence data with providers to bolster clinical care and decision-making. Reminder messages following detected nonadherence were perceived as a key feature, and customization was desired. Suggested improvements are primarily related to the design and usability of the Reader (a wearable device).

**Conclusions:**

Overall, individuals with HF perceived the DPS to be an acceptable and useful tool for measuring medication adherence. Accurate, real-time ingestion data can guide adherence counseling to optimize adherence management and inform tailored behavioral interventions to support adherence among patients with HF.

## Introduction

Heart failure (HF) is one of the leading causes of morbidity and mortality in the United States, affecting approximately 6.2 million Americans [1,2]. In 2018, a total of 13.4% of deaths in the United States were attributed to HF [2]. HF is also one of the most common causes of hospitalization in individuals aged 65 years or older [3]. Among those admitted to the hospital, nearly one-fifth will be readmitted to the hospital for complications related to HF or other comorbidities within 30 days [4-6]. Pharmacologic management of HF focuses on increasing uptake and adherence to goal-directed quadruple medical therapy: angiotensin receptor-neprilysin inhibitor, β-blocker, mineralocorticoid receptor antagonist, and sodium-glucose co-transporter 2 inhibitors [7]. This strategy has demonstrated high efficacy for reducing hospital readmission and progression of HF and its associated cardiometabolic outcomes [8,9].

Medication nonadherence is a leading driver of worsening clinical outcomes in HF. Large longitudinal cohort studies have demonstrated that nonadherence to any pillar of HF pharmacotherapy is associated with increased all-cause mortality and an increased risk of 30-day hospital readmissions [10,11]. In a large, single-center, cross-sectional study, up to 15% of hospital readmissions in individuals with HF were associated with medication nonadherence [12]. Additionally, in individuals admitted to the hospital, 28% experience primary nonadherence to a component of HF pharmacotherapy as short as 1 week after discharge, with 24% experiencing persistent nonadherence at 30 days [13]. Given the close relationship between nonadherence and hospital readmission among individuals with HF, it is critical to continue to develop techniques that allow for the assessment of medication adherence in this population [14-20].

**Figure 1:**
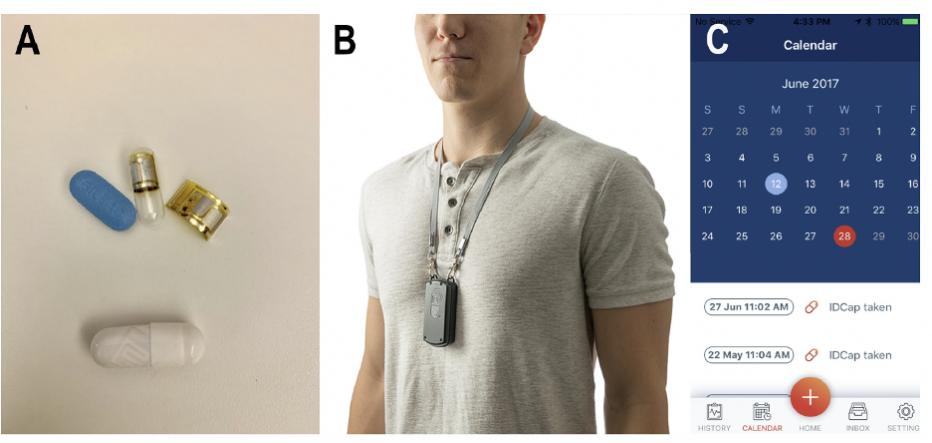
Components of the digital pill system (DPS; ID-Cap System; etectRx). (A) A radio frequency identification-tagged capsule with pill, (B) the Reader device worn on a lanyard over the neck, and (C) the smartphone app displaying the details of a digital pill ingestion.

Current strategies for measuring adherence to HF pharmacotherapy include pharmacy refills, as measured by the medication possession ratio, and the number of subsequent days the patient has access to medications, as measured by the proportion of days covered [21,22]. This approach assesses overall adherence over periods of time, yet it is suboptimal in its capacity to capture daily challenges to adherence that may ultimately affect overall adherence and HF outcomes [23]. In contrast, one strategy for directly measuring daily adherence is a digital pill system (DPS; Figure 1). DPS technology is comprised of a gelatin capsule with an integrated radiofrequency emitter that overencapsulates the desired medication. Following ingestion of the digital pill, the radiofrequency emitter is activated by gastric chloride ions, which then projects a unique radio signal off the body that is acquired by a wearable device (Reader) [24,25]. The Reader stores and forwards ingestion data through low-energy Bluetooth to the user’s smartphone and a clinician dashboard, enabling both patients and care teams to assess adherence patterns in real-time [26]. This strategy has been previously leveraged to measure oral pharmacotherapy adherence to antidiabetic and antihypertensive medications [27-30].

To understand potential user responses to the DPS and inform future research involving this technology among individuals with HF, we conducted brief quantitative assessments and semistructured qualitative interviews to explore perceived facilitators of and barriers to the use of a DPS that measures adherence to HF pharmacotherapy.

## Methods

### Participants

All participants met the following inclusion criteria: (1) aged 18 years or older, (2) admitted to inpatient general medical or cardiology services with a diagnosis of HF, and (3) currently on oral HF pharmacotherapy. Individuals were excluded if they (1) had a history of heart transplant, (2) had an implanted left ventricular assist device, (3) were non-English speaking, or (4) were admitted to an intensive care unit.

### Procedures

Participants were recruited in-person at a large, urban academic quaternary care hospital in Boston, Massachusetts, where patients with HF are admitted to either the general medical service or cardiology services; both inpatient teams independently manage patients with standardized treatment algorithms. Participants were not previously known to or in direct clinical care with any members of the study team. All study procedures were conducted in-person in a private area at the hospital while participants were inpatient.

Following verbal consent, participants completed a digitally recorded, semistructured qualitative interview with a bachelor’s-level research assistant (either male or female) trained in qualitative interviewing techniques (JJK and MC). Interviews ranged from 23 minutes to 64 minutes in length (mean duration of 39 minutes). We adhered closely to the Consolidated Criteria for Reporting Qualitative Research (COREQ) guidelines (Multimedia Appendix 1) [31]. Study staff explained the components and functionality of the DPS (ID-Cap System; etectRx) in detail. Debrief documents were written after each interview and shared with the study team, who assessed for thematic saturation. Participants also completed a brief quantitative assessment. Following the completion of all study visit procedures, remuneration was provided. Study procedures were completed from April to August 2022.

### Measures

#### Qualitative Interview

A qualitative interview guide (Multimedia Appendix 2) was developed by the study team members with expertise in the DPS, goal-directed medical therapy for HF, medication adherence, and technology development (PRC, JLS, MV, and BMS). Questions explored baseline adherence to HF medications and current adherence strategies, initial responses to DPS technology and messaging infrastructure through the DPS app or SMS text messaging, perceived facilitators of and barriers to DPS use, and perceptions of data privacy in the DPS context. Following an overview of the DPS technology and component parts, participants were asked whether they would be willing to use the DPS for HF adherence monitoring; this question was used to evaluate overall acceptance of the technology. The interview guide was piloted for completeness among members of the study team before implementation. Sample interview questions are provided in Table 1.

**Table 1:** Sample qualitative interview content areas, questions, and probes used during the study.

Content area	Sample probes
Current adherence strategies	How long have you been prescribed a diuretic or SGLT2i^a^? How have you tried to remember to take your medications? What kind of barriers do you face to taking your medications on time?
DPS^b^ technology	What are your initial reactions to the digital pill? Are there design factors to the digital pill and Reader that prevent you from wanting to use it? Why would these factors prevent your use of digital pills?
DPS messaging components	Tell me about situations you would like to receive notifications about your adherence. What kind of messages would you want to receive in relation to the digital pill?
Data privacy and sharing	The digital pill allows your provider or study team to view your adherence. What do you think of this? What concerns do you have regarding the privacy of your adherence data? Who do you think should have access to your adherence data? Why?
Acceptance of and willingness to use the DPS	Given what you know, would you be willing to use the digital pill? Why or why not?

^a^SGLT2i: sodium-glucose co-transporter 2 inhibitors.

^b^DPS: digital pill system.

#### Quantitative Assessment

Quantitative assessments collected data surrounding sociodemographics and HF history. Participants were asked to estimate their adherence to HF medications over the past 3 months on a 0% to 100% sliding scale. We also provided a list of common medication adherence systems (eg, pill boxes, automated phone reminders, and smartphone apps) to assess previous use of such adherence strategies. These questionnaires were developed by the study team, which also supervised participants in completing the baseline assessment.

We used 3 subscales of the previously validated Media Technology Usage and Attitudes Scale (MTUAS) to measure attitudes toward technology: the positive attitudes subscale (6 items, eg, “With technology anything is possible”), the negative attitudes subscale (3 items, eg, “New technology makes life more complicated”), and the anxiety or dependence on technology subscale (3 items, eg, “I get anxious when I don’t have my cell phone”) [32]. Items were rated on a 5-point Likert scale (1=strongly disagree and 5=strongly agree). Score ranges were 1-5 for each subscale, with higher scores indicating more positive attitudes, more negative attitudes, and more technological anxiety and dependence [32]. The final quantitative assessment was cognitively tested among the study team to ensure clarity of questions before deployment with participants.

### Analyses

Descriptive statistics were calculated to characterize the sample. Qualitative interviews were professionally transcribed and scrubbed of identifiers. Applied thematic analysis was used to code and analyze the interviews [33]. As part of the applied thematic analysis approach, 3 study team members (JJK, JJT, and GRG) reviewed all interview transcripts in order to iteratively generate a coding framework using a combination of the interview guide questions and data from the interviews themselves. Parent codes and subcodes were iteratively added to the coding framework throughout the transcript review process, and the final coding framework was then reviewed and revised by the study team before the formal coding of transcripts for the purpose of identifying qualitative domains and themes. Our 2 independent coders (JJK and JJT) double-coded 25% of the transcripts to establish interrater reliability; a κ score of >0.8 was used to establish adequate reliability between the coders, and this threshold was met. Study team members (JJK, JJT, and GRG) reviewed and compared coding throughout this process to discuss and resolve discrepancies, with oversight from the study’s principal investigator (PRC). Following the resolution of all coding discrepancies in double-coded transcripts, the coders (JJK and JJT) then independently coded the remaining 75% of transcripts. An audit trail of computerized coding was maintained. Salient quotes from the interviews were extracted, discussed with a subset of the study team (JJK, JJT, PRC, and GRG) to identify major domains and themes, and then disseminated to the entire study team for review. Coding was facilitated by NVivo software (QSR International).

### Ethical Considerations

All study procedures were approved by the Mass General Brigham Institutional Review Board (2022P000545). We obtained written informed consent from all study participants. Study data were anonymized, and all study participants were only identified by a unique study identification number. Transcripts of interviews were scrubbed of any identifiers before analysis. Participants were compensated US $40 at the completion of interviews.

## Results

### Participant Characteristics

Over the study period, 96 individuals met the inclusion criteria. Of these, 43 (45%) were discharged before they could be approached by the study team. Of the remaining 53 individuals, 12 (23%) were unavailable for consent, and 21 (40%) declined to participate. The reasons provided for declining participation included the time commitment for study procedures (n=2), general lack of interest (n=10), lack of knowledge of current medications (n=1), perception that they did not match the target study population (n=1), dissatisfaction with current clinical care (n=1), and reason unknown (n=6). A total of 20 participants consented and completed all study procedures (mean age 68, SD 14.3 years). The sample was predominantly female (n=11, 55%), White (n=13, 65%), and non-Hispanic (n=18, 90%). Full sociodemographic information is provided in Table 2.

**Table 2:** Sociodemographic characteristics of study participants (n=20).

Variable		Value
Age (years), mean (SD)		68 (14.3)
**Sex, n (%)**		
	Male	9 (45)
	Female	11 (55)
**Race, n (%)**		
	Black or African American	6 (30)
	White	13 (65)
	Other	1 (5)
**Ethnicity, n (%)**		
	Hispanic or Latino	2 (10)
	Not Hispanic or Latino	18 (90)
**Education, n (%)**		
	High school graduate or GED^a^	2 (10)
	Some college	6 (30)
	College degree	8 (40)
	Some graduate school	2 (10)
	Graduate or professional	2 (10)
**Annual income (US $), n (%)**		
	6000-11,999	4 (20)
	12,000-23,999	3 (15)
	24,000-29,999	2 (10)
	30,000-$59,999	3 (15)
	≥60,000	8 (40)

^a^GED: general educational development.

### Quantitative Results

Half (10/20, 50%) the sample had HF with preserved ejection fraction, and the other half (10/20, 50%) had HF with reduced ejection fraction. Most (11/20, 55%) were diagnosed with HF over 5 years ago, and half (10/20, 50%) had been admitted to the hospital multiple times due to HF in the past year. All (20/20, 100%) expressed at least some degree of concern regarding worsening HF. Self-reported adherence during the previous 3 months was high (mean 90.1%, SD 17.1%), and most (12/20, 60%) reported using a system to maintain adherence, with a standard pill box as the most common strategy (10/20, 50%). Finally, most participants (11/20, 55%) reported that visualizing their individual adherence patterns would motivate them to maintain adherence. Full HF status and pharmacotherapy adherence data are presented in Table 3.

**Table 3:** Heart failure (HF) status and pharmacotherapy adherence among study participants (n=20).

Variable							Value
**HF status**							
	**Duration of HF (years), n (%)**						
					<1	1 (5)	
					1-2	2 (10)	
					2-5	6 (30)	
					>5	11 (55)	
	**Primary physician managing HF treatment, n (%)**						
					Cardiologist	13 (65)	
					Primary care physician	2 (10)	
					Does not know	4 (20)	
					Other	1 (5)	
	**Number of prescribed HF medications, n (%)**						
					1	1 (5)	
					2-5	10 (50)	
					>5	9 (45)	
	**Type of HF, n (%)**						
				HFpEF^a^		10 (50)	
				HFrEF^b^		10 (50)	
	**Number of hospital admissions for HF over the last 12 months, n (%)**						
			0			2 (10)	
			1			7 (35)	
			2-5			10 (50)	
			>5			1 (5)	
	**Number of physician encounters due to concerns surrounding worsening HF over last 12 months, n (%)**						
				0		7 (35)	
				1-5		10 (50)	
				6-10		1 (5)	
				11-20		2 (10)	
	**Degree of concern about HF, n (%)**						
			Slightly concerned			2 (10)	
			Moderately concerned			5 (25)	
			Very concerned			4 (20)	
			Extremely concerned			9 (45)	
**Pharmacotherapy adherence**							
	Percentage of self-reported HF medication adherence over last 3 months, mean (SD)					90.1 (17.1)	
	**Uses a system to maintain medication adherence, n (%)**						
			Yes			12 (60)	
			No			8 (40)	
	**Medication adherence systems used, n (%)^c^**						
			Smart pill box			1 (8)	
			Pill organizer			10 (83)	
			Smartphone-based reminders			3 (25)	
			Other			1 (8)	
	**Visualization of adherence patterns would motivate medication adherence, n (%)**						
		Yes				151 (55)	
		No				7 (35)	
		Unsure				2 (10)	
	**Willingness to use the DPS^d^** **, n (%)**						
		Yes				12 (60)	
		No				8 (40)	

^a^HFpEF: heart failure with preserved ejection fraction.

^b^HFrEF: heart failure with reduced ejection fraction.

^c^Participants were provided with the opportunity to select multiple options, if applicable.

^d^DPS: digital pill system.

In terms of technology usage, three-quarters (15/20, 75%) of the sample owned a smartphone. MTUAS scores indicated positive attitudes toward technology (mean 4.2, SD 1.1) and a moderate degree of anxiety around being without technology and dependence on technology (mean 3.3, SD 1.4). Technology usage and MTUAS scores are provided in Table 4.

**Table 4:** Technology usage and the Media Technology Usage and Attitudes Scale (MTUAS) scores among study participants (n=20).

Variable						Value
**Technology usage, n (%)**						
	**Owns a smartphone**					
			Yes		15 (75)	
			No		5 (25)	
	**Ever used a smartphone to communicate with medical care team**					
		Yes			13 (65)	
		No			7 (35)	
	**Methods used to communicate with medical care team using a smartphone^a^**					
				Phone call	12 (92)	
				Through hospital portal (Patient Gateway)	10 (77)	
				Email	8 (62)	
				SMS text message	6 (46)	
				Other	2 (15)	
**MTUAS, mean (SD)**						
	Positive attitude toward technology subscale score				4.2 (1)	
	Negative attitude toward technology subscale score				3.0 (1)	
	Anxiety or dependence on technology subscale score				3.3 (1)	

^a^Participants were provided with the opportunity to select multiple options.

### Qualitative Results

Key findings surrounding the use of DPS technology for HF pharmacotherapy adherence emerged across the following major domains: (1) initial responses to the DPS, perceived barriers to use, and overall willingness to use the technology; (2) perceptions around privacy and sharing of DPS data; (3) responses to DPS messaging components; and (4) suggested improvements for future iterations. Multiple themes emerged within each domain; these are discussed in detail below.

#### Initial Responses, Perceived Barriers, and Overall Willingness to Use the DPS

Most participants perceived the DPS to be a novel, reliable tool for adherence measurement. They described the real-time data it generates as potentially useful for reinforcing adherence behavior and noted that it would increase their sense of personal accountability for their HF regimen. Importantly, many participants described instances in which they were unsure whether they had taken their medications for the day and viewed the DPS as a valuable means for confirming past medication ingestions to avoid double dosing; this was interpreted as an indication of participants’ perceived usefulness. After learning about the DPS, 60% (12/20) participants indicated a willingness to use the DPS to measure their HF pharmacotherapy adherence.

Absolutely I would use it. Because it’s easier...It would help a whole lot. Because it would show [my physician] when or if I was adhering to the protocol. He’d know I’m taking my medicine or if I’m not.

Aged 59 years, male

I think it would be great—like a 30-day regime, make sure we’re all on the same book kinda thing...If I was older, or I was gettin’ blinky, or I didn’t have caretakers or people looking out for me, it wouldn’t be a bad idea.

Aged 67 years, female

I think it could be very useful for some people, and I doubt that I would use it right now in my present level of decline. But if I start having memory problems or if I ever start having problems taking medication, I’d be very interested in it.

Aged 80 years, male

Participants also identified a number of key barriers to DPS use. These included the perceived complexity of operating the technology, which was particularly salient among individuals who did not own smartphones. Some participants also described the Reader as large and potentially stigmatizing in the event that they needed to use the DPS in public. For some participants, the presence of electronics within the digital pill itself (ie, the radiofrequency emitter) raised questions around safety; however, most of these concerns were mitigated after participants were informed that the DPS in question (ID-Cap System; etectRx) had received Food and Drug Administration (FDA) clearance for use in humans. Other reported barriers to DPS uptake included potential costs associated with the device and a general lack of need for adherence support.

I don’t have a comfort zone with technology. It scares me because I tried to learn, you know, particularly the phone, and I just get nervous...if I pick up something like that and do it, my mind just shuts down.

Aged 80 years, male

It does become a problem because you’ve probably already got everything else charged, and then you have to find a plug, figure out where you’re gonna go with it. Then, if you got little kids, it’s like, “What’s that?” A whole bunch of headache.

Aged 46 years, female

#### Perceptions Around Privacy and Sharing of DPS Data

Some participants reported privacy-related concerns, including a fear of unwarranted tracking or interdiction of their adherence data and the potential for tampering with data, as additional barriers to DPS use. In particular, these participants expressed worries about whether swallowing a pill containing a radiofrequency emitter could transmit unwanted personal information to others related to their medications, adherence behavior, location tracking, and other physiological data.

You’re gonna have to sit in front of me and explain to me how it’s secure. What is making that radio frequency secure? Because I’m not just gonna randomly believe somebody that says, “Oh, well, you’re gonna swallow this magic pill. It’s gonna have a motherboard inside and it’s gonna randomly broadcast to an outside device, and tell people what medications you’re on, what you’re taking, when you’re taking it—and potentially additional information about it.”

Aged 58 years, female

Despite expressing some concerns around data transmission and privacy, overall, participants expressed a desire for their DPS adherence data to be shared with their clinical care teams, given its importance for preventing the progression of HF. They reported that sharing DPS data with providers would be more reliable than self-reporting adherence and that it could be used to guide conversations around medication side effects, additional adherence or behavioral support that may be needed, and adjustments to medication regimens, including in the setting of worsening disease. Other participants shared more mixed opinions; while these individuals were willing to share adherence data with providers, they were unsure if doing so would meaningfully impact their ongoing HF treatment.

If a doctor looks at it and sees that you’re not taking your medication, well, of course, something’s gonna have to be done...There’s nothing bad about the data going to the doctor...it’s all positive. It certainly can’t hurt.

Aged 71 years, male

It’s so important to let your physician know you’re actually taking that medication as prescribed. So if something is not working, then they know there’s no question that this person was adhering to the prescribed treatment. And maybe this medication is not working for them. Maybe they need to increase it or get another one.

Aged 62 years, female

#### Responses to DPS Messaging Components

Participants were presented with an overview of three types of messages that can be programmed within the DPS: (1) confirmatory messages, sent after each ingestion to indicate successful detection; (2) reminder messages, sent before a prespecified dosing window; and (3) nonadherence reminder messages, sent after a dosing window if no ingestion had been detected.

Most participants accepted confirmatory messages following ingestions and viewed them as a useful feature for instances in which they were unsure if they had correctly operated the DPS. Importantly, because HF pharmacotherapy consists of multiple medication regimens, participants emphasized the need for confirmatory messages to specify the name of the medication ingested. They also expressed a desire to customize the timing of confirmatory messages; while some preferred a confirmation after each ingestion, others preferred less frequent messages, such as only at the end of the day or the week, as part of an adherence summary. Relatedly, participants suggested that the frequency of messages could increase or decrease over time, based on DPS-detected patterns of adherence and nonadherence.

I found the [confirmatory messages] a little annoying. I get too many text messages, so where it would be helpful is if I’d forgotten to take the medicine, then if I got a reminder in a text message to take my medicine, that would be great. Once I’ve done it, I don’t need the confirmation.

Aged 80 years, male

Overall, the majority of participants viewed reminder messages—and in particular, reminder messages that follow nonadherence detected by the DPS—as one of the most important features of the technology. Most reported that changes in routine and forgetfulness were common reasons for missed doses and noted that just-in-time reminders would be helpful for maximizing the potential for adherence in the moment, as well as for positive reinforcement around adherence behavior more generally. Some participants also noted that it would be useful to integrate their existing reminder systems, such as smartphone alarms, into DPS-based reminder messages in order to further reinforce adherence.

Usually what happens is, I don’t know until the next day that I forgot to take [my medications], whereas, if I got a reminder at 9:00 p.m. saying, “Hey, you didn’t take your nightly pills,” that would be better, because then I could go take them.

Aged 82 years, male

So you don’t need to beat somebody over the head, but they need to be told, “You missed your Lasix. This is a problem. You know, if you keep missing your Lasix, you could end up in the hospital.” Like it needs to be made clear.

Aged 58 years, female

Participants also expressed an interest in customizing both the timing and content of reminder messages. In terms of timing, participants largely preferred a maximum of 2 messages proximal to each dosing window—for example, a reminder 30 minutes before the window and a follow-up reminder 15 minutes after the window if no ingestion was detected. Regarding the content of nonadherence reminders, participants reported an interest in simple messages indicating that they had forgotten to take their medication. Some also suggested that reminder messages could represent an opportunity to deliver HF-related educational information, especially related to the consequences of medication nonadherence.

You could do a snooze. You can pick, “Okay. Remind me five minutes before or five minutes after and during.” I don’t know. But let the person be able to choose how many reminders that they get.

Aged 40 years, female

If on the app, there’s a little alarm that goes, “Hey, dummy, it’s time to take your pill,” and then I take a pill, and it monitors me taking the pill, then that’s pretty much all you could ask.

Aged 63 years, male

#### Suggested Improvements for Future Iterations

Most recommendations focused on technological and design-based improvements to the Reader that would improve the user experience. Suggested enhancements included a new form factor that could integrate into typical clothing (eg, a pocket clip, wristband, smartphone case, or necklace). Participants also suggested that integrating additional features into the Reader, such as a voice assistant and colored lights to indicate adherence and reminders, would be helpful for individuals who do not carry a smartphone. Customization of the exterior casing of a Reader was also proposed, as was a stand-alone device that could provide adherence feedback independent of a smartphone. Finally, participants emphasized that future iterations of the system should come with detailed information around security protections and clear instructions for use.

I’d rather put it in my pocket or hold it in my hand. The best is just being able to plug it in and forget it...just because it’s something you don’t have to worry about anymore. I mean I have things plugged in around my house...I don’t think anything about ’em—they’re doing their job and that’s all I have to do.

Aged 82 years, male

In the directions, I would want to be told that it’s not harmful and why it’s not harmful...I would like to know how long this is [for]. Like the directions say, if you take this gelatin pill...it will stay inside you for three months and it’ll help us to track this for three months. I would like very clear instructions on how to use it.

Aged 58 years, female

## Discussion

### Overview

HF is one of the leading causes of hospital readmissions and mortality in the world [1,2]. One key pillar in efforts to optimize medical management of HF includes maximizing adherence to pharmacotherapy. While DPS technology has previously been shown to accurately measure medication adherence across a wide spectrum of diseases, its efficacy has yet to be described in the context of HF treatment [27-30]. This qualitative investigation provides formative data surrounding the acceptance and design of a DPS that directly measures HF pharmacotherapy adherence. Findings indicate that participants were accepting of the DPS overall and perceived the system as a tool for enhancing accountability and providing data to inform the ongoing medical management of HF. Personalized adherence reminders were identified as a key component of the system. These data demonstrate the potential for DPS deployment to measure adherence among individuals with HF.

### Principal Findings

After learning about the DPS, 60% (12/20) of participants perceived the system as an “acceptable” strategy to measure HF pharmacotherapy adherence. Participants also expressed the “usefulness” of the DPS based on its perceived ability to motivate adherence, provide accountability, and avoid double-dosing. For most individuals, having incontrovertible evidence of their adherence (or nonadherence)—especially in the context of clinical care, where their DPS adherence data could aid discussions with their HF physicians and guide future medication decisions—was perceived as the most valuable benefit. These qualitative findings reinforce other proposed benefits in the literature of leveraging real-time adherence data to not only address medication adherence in chronic disease but potentially enhance the patient-physician relationship by providing key data to ground conversations surrounding disease progression [34]. This concept was reflected in the quantitative portion of the study, where 55% (11/20) of individuals considered having a visual record of ingestion patterns over time as a motivating factor to continue to maintain medication adherence. These perceptions are consistent with other investigations that suggest individuals with other chronic diseases find value in DPS-based adherence data as a technique to guide pharmacotherapy [35-37]. Together, our data suggests that future research investigations should seek to understand the feasibility of real-world DPS operation among individuals with HF, as well as evaluate the impact of adherence metrics on disease progression and treatment regimens in both research and clinical deployment contexts.

Efforts to optimize the design of DPS technology to measure adherence to HF medication should also include a customizable messaging architecture that responds to detected patterns of adherence. Based on our data, messaging components should include confirmation messages to help individuals recognize that they correctly operated the DPS and recorded their medication ingestion. Most importantly, messaging modules should include nonadherence reminder messages that respond to adherence patterns from the DPS, which participants identified as a critical and valuable component of the system. A major theme that emerged from our interviews was participants’ desire for control over both the timing and content of reminder messages related to their adherence patterns. While some wanted daily or even more frequent messaging that would coach them through adherence lapses, others preferred only on-demand access to their adherence data and less frequent feedback from the system. Importantly, some participants also reported that reminder messages could represent a potential method for providing educational information about HF and reinforcing DPS users’ understanding of the consequences of medication nonadherence. These emerging themes demonstrate the importance of involving patients in the design and delivery of adherence interventions linked to digital health systems such as the DPS [29,38]. Barriers to the use of the DPS included discomfort with technology among some users and concerns about the privacy and security of their data.

Ultimately, participants viewed the DPS in its current iteration as usable, but they suggested several key improvements that would enable better integration into daily life. Some of these suggestions, including miniaturizing the Reader and providing alternative off-body systems that can collect adherence data, are currently under investigation in other ingestible sensor trials [39]. Additionally, participants expressed that DPS deployment should only occur alongside a detailed discussion with users about the safety and security of the system. While the DPS is FDA 510k cleared, participants emphasized the importance of providing users with data from past users of the system, particularly surrounding any DPS-related adverse events [25].

### Limitations and Future Studies

This study had several limitations. First, the sample consisted of a small number of inpatients recruited as part of a convenience sample at a single hospital site. Qualitative data around patient experiences with HF and responses to DPS technology may vary across other health care institutions and patient populations. Second, perspectives from non–English-speaking individuals are missing, as this study only enrolled English-speaking participants; future investigations should explore responses to DPS technology in non-English speakers. Third, qualitative interviews explored perceptions of the technology among participants who did not ingest any digital pills or use the DPS themselves. The lived experiences of participants who use and operate the DPS in a clinical trial setting may differ.

### Conclusions

In conclusion, this study demonstrates that individuals with HF perceived DPS technology to be an acceptable and useful tool for measuring medication adherence, informing our understanding of how this technology can be operationalized with this patient population in the real world. Importantly, this investigation also defined key boundary conditions for the physical design of the DPS as well as the structure of reminder messages that both support adherence and confirm the correct operation of the DPS. Finally, these formative data will help to inform best practices for future studies that develop interventions to support HF pharmacotherapy adherence and assess the efficacy of the DPS in this context.

## Appendix

Multimedia Appendix 1 COREQ (Consolidated Criteria for Reporting Qualitative Research) checklist.

Multimedia Appendix 2 Qualitative interview guide.

## Abbreviations

COREQ: Consolidated Criteria for Reporting Qualitative Research
DPS: digital pill system
FDA: Food and Drug Administration
HF: heart failure
MTUAS: Media Technology Usage and Attitudes Scale

## Data Availability

The data sets generated during and/or analyzed during this study are available from the corresponding author on reasonable request.
