# Supplementary material for: Formative Perceptions of a Digital Pill System to Measure Adherence to Heart Failure Pharmacotherapy: Mixed Methods Study
Source: JMIR Cardio. 2024 Feb 15;8:e48971. doi: 10.2196/48971 (PMC10905352; doi:10.2196/48971)
Supplement: Multimedia Appendix 2 [file cardio_v8i1e48971_app2.docx]

Formative acceptance of digital pill systems to measure medication adherence in individuals with heart failure

Aim 1 Semi-structured interview agenda

V1.0; February 21, 2022

*The intent of these individual interviews is to help gather preliminary data on the acceptance of digital pills to measure adherence to SGLT2i and furosemide among individuals with heart failure (HF). This agenda is intended to guide study staff through the key content areas of data collection for this project, ensuring that the same content is discussed in each semi-structured interview. This agenda serves as a guide to the study staff, not a rigid script that must be adhered to verbatim. This allows the study staff flexibility to adapt and clarify questions to suit the needs of different study subjects. Similarly, questions need not be asked in this particular order. Instead, the study staff will adapt the conversation as needed according to the narrative from each study subject, pursuing both the research topics as well as any relevant emergent themes that evolve from the discussion.*

1. **Welcome**

*Intent: The goal of section A is to welcome and consent study subjects, and explain the purpose of this interview.*

1. Introduce yourself
2. Explain that we will be recording the interview so we can analyze themes from their stories later and that
   1. All recorded data is in no way linked to the study subject
   2. We will not be using their name in the recording
3. Study subjects are the only ones who can help us better understand how best to refine and use of digital pills as a measure of adherence.
4. Of note, participants will NOT be asked to swallow any digital pills during the interview.
5. Remind subjects to not use their names during the recording. If this occurs during the recording, remind participants that we will delete their name or other identifiers from the interview transcript.
6. Answer any questions

**B. Baseline Adherence**

*Intent: The goal of section B is to assess the degree of adherence to current medications that participants have, and identify any pre-existing methods participants use to reinforce adherence. In particular, the interviewer should focus on the participant’s use of diuretics (furosemide, bumetanide, torsemide) and SGLT2 inhibitors (empagliflozin, dapagliflozin, canagliflozin)*

1. How long have you been prescribed a diuretic or SGLT2i?
2. Why do you think your doctor asked you to take this? How did it feel to be told you need to take a medication to manage your heart failure?
3. Why do you think it is hard to take medications as prescribed? Do you have difficulties doing this? Which medications?
   1. How do you feel when you’re taking your medications on time? When you aren’t?
4. How have you tried to remember to take your medications?
5. Do you use any technology-based reminder systems?
6. Have you used any technology-based reminder systems in the past? What do you think of them?
7. Do you have a smartphone? What type/operating system?
8. What kind of barriers do you have to taking medications on time?
   1. Probe: Do you have issues around refilling medications?
   2. Probe: How do you feel when you find out that you’ve forgotten or delayed taking your medication?

**C. Digital pill technology**

*Intent: The goal of section C is to gain insight from participant about digital pills. We are interested in how they perceive the technology, how they envision interacting with it, and how they would design it to improve its acceptance amongst their peers. During this section, we will show participants mock-ups of the digital pill and its components and allow participants to handle the devices.*

1. Introduce study participant to the digital pill system and Reader. Explain how the system works, the intent of using digital pills (adherence monitoring), and potential use of feedback from the digital pill.
2. What are your initial reactions to the digital pill?
3. Are there design factors to the digital pill and Reader that prevent you from wanting to use it?
   1. Probe: (if participants identify components of the digital pill that prevent them from wanting to use it) Why would these factors prevent your use of digital pills?
   2. Any ways you can think of to get around this issue?
4. Show the participant the reader: what are your thoughts about wearing the Hub?
   1. Any changes to its design that would help?
   2. What do you think about integrating the technology into another device that you use daily (eg. watch, smartphone)?

**D. Messaging and the digital pill.**

*Intent: The goal of section D is to determine the optimal timing and style of messaging that is delivered from the digital pill in response to adherence and nonadherence.*

1. If you were using the digital pill to help measure your adherence to your heart failure medication, what would be the most effective way for us to help you take your medication on time?
2. We typically program the digital pill to send you a confirmatory text message each time you took your digital pill. Is that too much? Do you want confirmation? Why/why not?
   1. What is the optimal number of times you would want ingestion confirmation messages? Is it every day, every other day, or another frequency? Why?
3. Tell me about situations you would like to receive notifications about your adherence?
4. What kind of messages would you want to receive in relation to the digital pill?
   1. Probe: would you want to receive reminders every time you missed a dose?
   2. Probe: how would it feel for you to get feedback around missing pills?
5. Would you prefer to have a companion app on your smartphone that would display your adherence data in real time to you, or do you think text messaging is enough?
6. Would you be willing to receive messages regarding other influences of adherence?
   1. Probe: for example, if your diuretic or SGLT2i makes you urinate, would you want reminders to help you time your medication use around your other daily activities?
7. What is the best way for you to have messages displayed? Simple text, images, videos, or a combination?

**E. Privacy and practices.**

*Intent: The goal of section E is to discuss the implications real-time adherence monitoring has on participant’s perceptions of privacy. Additionally, we are interested in understanding what it means for participants to view adherence data from digital pills.*

1. The digital pill allows your provider or a study team to view your adherence. What do you think of this?
   1. What concerns do you have regarding the privacy of your data?
   2. Would digital pills make you want to become more adherent to your HF medication? Why/why not?
   3. Who do you think should have access to adherence data? Why?
2. Tell us your thoughts on what it means for others to see your adherence data?
   1. Would you be comfortable showing people that you are on HF medication? That you are adherent/nonadherent?
   2. How would it feel to show others your adherence data? Physicians? Family members? Others?

**F. Future recommendations**

*The goal of section E is to gather information regarding how study participants would design digital pills and deliver the message of its intended use to maximize its acceptance among individuals.*

1. Given what you know, would you be willing to use the digital pill? Why/Why not?
2. If you were introducing the digital pill to your friends, what would you say about it?
3. What do you think are the major barriers that people face with digital pills?
4. What are potential factors that would prevent someone from wanting to use this technology?
5. What aspects of the technology would make you want to use the digital pill more?

**H. Closing and follow up**

Thank you for participating in this interview. The recording will now end. We appreciate all the information and feedback you have given us.

1. Answer any lingering questions
2. Turn off voice recorder
3. Provide participant with compensation
